# Genome‐wide screen for anticancer drug resistance in haploid human embryonic stem cells

**DOI:** 10.1111/cpr.13475

**Published:** 2023-04-21

**Authors:** Emanuel Segal, Jonathan Nissenbaum, Mordecai Peretz, Tamar Golan‐Lev, Rivki Cashman, Hagit Philip, Benjamin E. Reubinoff, Oded Kopper, Nissim Benvenisty

**Affiliations:** ^1^ The Azrieli Center for Stem Cells and Genetic Research, Department of Genetics, Silberman Institute of Life Sciences The Hebrew University Jerusalem Israel; ^2^ Hadassah Stem Cell Research Center, Goldyne Savad Institute of Gene Therapy, Department of Obstetrics and Gynecology Ein Kerem, Hadassah Hebrew University Medical Center Jerusalem Israel; ^3^ NewStem LTD Jerusalem Israel

## Abstract

Anticancer drugs are at the frontline of cancer therapy. However, innate resistance to these drugs occurs in one‐third to one‐half of patients, exposing them to the side effects of these drugs with no meaningful benefit. To identify the genes and pathways that confer resistance to such therapies, we performed a genome‐wide screen in haploid human embryonic stem cells (hESCs). These cells possess the advantage of having only one copy of each gene, harbour a normal karyotype, and lack any underlying point mutations. We initially show a close correlation between the potency of anticancer drugs in cancer cell lines to those in hESCs. We then exposed a genome‐wide loss‐of‐function library of mutations in all protein‐coding genes to 10 selected anticancer drugs, which represent five different mechanisms of drug therapies. The genetic screening enabled us to identify genes and pathways which can confer resistance to these drugs, demonstrating several common pathways. We validated a few of the resistance‐conferring genes, demonstrating a significant shift in the effective drug concentrations to indicate a drug‐specific effect to these genes. Strikingly, the p53 signalling pathway seems to induce resistance to a large array of anticancer drugs. The data shows dramatic effects of loss of p53 on resistance to many but not all drugs, calling for clinical evaluation of mutations in this gene prior to anticancer therapy.

## Graphical abstract

Genome‐wide mutation library screens were performed to identify anticancer drug‐resistance genes through exposure of haploid human embryonic stem cells to anticancer drugs, identifying and subsequently validating enriched genes as conferring drug resistance.

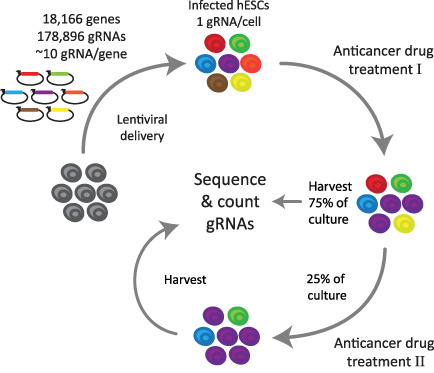

## 1 INTRODUCTION

Chemotherapies and other anticancer drugs stand at the forefront of cancer therapy, eradicating tumours and assisting in prolonging patient survival.^1^, ^2^, ^3^ Nevertheless, tumours frequently show resistance to treatment, either innate or acquired following initial treatment. Resistance to anticancer drugs is a leading cause of ineffectual cancer treatment and tumour relapse, leading to poor survival rates.^4^, ^5^ The identification of genes and pathways that confer drug resistance is therefore of prime interest in the field of cancer research. The application of drug treatments to a wide panel of human cancer cell lines (hCCLs), followed by examining their response and gene expression, have yielded valuable insights regarding drug potency and mechanism.^6^ However, a direct interpretation linking a specific gene to drug resistance is not trivial. A forward genetic screening approach was suggested and applied as a powerful tool to identify genes and pathways responsible for or affecting a phenotype, requiring an efficient gene interference platform. Although gene knockdown screens by using RNA interference libraries (RNAi or shRNA) provided valuable data,^7^, ^8^, ^9^ they suffer from inherent limitations such as inconsistent RNA knockdown and a relatively high degree of off‐targets.^10^ With the recent advancement of CRISPR/Cas9 technology,^11^, ^12^, ^13^, ^14^ the ability to carry out comprehensive loss‐of‐function (LoF) screens not only sheds light on fundamental biological principles but also offered an attractive tool for drug target identification.^15^ The versatile nature of the CRISPR/Cas9 tool was applied to a large number of hCCLs for the identification of cancer‐essential genes and their correlation to anticancer drug response.^16^ However, a well‐known characteristic and limitation of hCCLs is their complex genomic profile. Aneuploidy/polyploidy, large genomic rearrangements, and a high rate of mutations, could easily mask gene/pathway relevance of essential genes.^17^, ^18^ KBM7 and subsequent derivative HAP1^19^ are near‐haploid (i.e., possessing a single copy of nearly all chromosomes) cancer‐derived cell lines that serve a valuable role in overcoming the polyploid genetic background,^20^, ^21^, ^22^ however they still show an abnormal genetic background and chromosomal instability^23^ due to their cancerous origin. A potential way of bypassing undesirable confounding interactions from other gene mutations is use of normal, genetically stable, human pluripotent stem cells (hPSCs) as a cancer‐related model. Both cancer cells and pluripotent cells share similarities in pivotal cellular features, making hPSCs a useful tool for cancer research.^24^, ^25^, ^26^

In the past decade, tremendous progress in mammalian forward and reverse genetics has been made by the introduction of mouse haploid embryonic stem cells,^27^, ^28^ followed by the generation of rat,^29^ monkey,^30^ and recently, haploid human embryonic stem cells (hESCs^31^, ^32^). The generation of the normal human cells with a single allele of each gene has opened exciting avenues for basic as well as applied research in human genetics.^18^, ^33^, ^34^, ^35^, ^36^, ^37^, ^38^

Here, we aim to exploit haploid hESC advantages as a tool for identifying anticancer drug resistant genes. The main advantages of a haploid hESC platform are efficacy and the normal genomic background, as the efficacy of genetic targeting depends on the number of target loci that need to be manipulated. If a cell line possesses extra copies of a given gene—a common occurrence in cancers—by probability, gene knockout will be more effective in haploid cells with a single copy. Furthermore, haploid hESCs have a normal genome with no effective point‐mutations,^31^, ^39^ and thus confounding mutations common in cancer cell lines can be avoided. To achieve a comprehensive perspective on mechanisms that might drive or regulate resistance to anticancer drugs, we performed genome‐wide LoF screens using a wide range of drugs. We selected 10 different anticancer drugs which target fundamental cellular mechanisms and are used for a broad range of cancer indications. Furthermore, our broad view of resistance‐related genes also illuminates an interesting and relevant aspect for the role that the *TP53* gene may play in chemoresistance. Collectively, our results emphasize the advantage of using haploid cells for wide LoF screens and the informative nature of using normal pluripotent cells for cancer research.

## 2 MATERIALS AND METHODS

### 2.1 Cell lines and culture

The following cell lines were used in this study: Haploid hESCs^31^ and h‐pES10‐based mutant library recently established by us.^18^ Diploid hESC‐TP53 LoF mutation with GFP‐tagged tubulin—TUB::GFP.; female 293T cells, obtained from R. Weinberg (Whitehead Institute). The library of mutated hESC were cultured at 37°C and 5% CO_2_ on matrigel‐coated plates (Corning) in feeder‐free mTeSR1 (STEMCELL Technologies) medium supplemented with 10 μM ROCK inhibitor Y‐27632 (Stemgent) for 1 day after splitting. Before reaching confluency, cells were passaged by a quick trypsinization using TrypLE Select (Thermo Fisher Scientific), plated in feeder‐free conditions. WA09 hESCs were cultured on feeder layer growth‐arrested mouse embryonic fibroblasts (MEFs) in standard hESC growth medium, composed of knockout Dulbecco's modified Eagle's medium (DMEM) supplemented with 15% knockout serum replacement (Thermo Fisher Scientific), 2 mM L‐glutamine, 0.1 mM nonessential amino acids, 50 units/mL penicillin, 50 mg/mL streptomycin, 0.1 mM β‐mercaptoethanol and 8 ng/mL basic fibroblast growth factor for maintenance and during the generation of knockout cell lines. MEFs and 293T cells were cultured in DMEM supplemented with 10% fetal bovine serum (Invitrogen), 2 mM L‐glutamine, 50 units/mL penicillin and 50 mg/mL streptomycin. All hESCs lines in this study, were used under the Israeli guidelines concerning hESC research (http://bioethics.webcare.org.il/english/report1/Report1-e.html).

### 2.2 Evaluation of hESC and hCCL anticancer drug response

To evaluate the correlation of different drugs' potency between hESCs and hCCLs, we measured hESCs' response to Approved Oncology Drug (AOD) Set collection of the NCI/DTP Open Chemical Repository Collection—a defined set of FDA‐approved anticancer drugs to enable cancer research (http://dtp.nci.nih.gov/repositories.html), and compared it with the response of CCLs as published in the NCI‐60 Growth Inhibition Data of the NIH (https://dtp.cancer.gov/discovery_development/nci-60/default.html). For each of the tested drugs, hESCs were treated with 20 μM and were measured with CellTiter‐Glo® Luminescent Cell Viability Assay 3 days later. We next ranked the different drugs according to their potency to induce cell death in both hESCs and hCCLs. The drug with the highest potency was ranked with the lowest viability score, whereas the drug with the lowest potency was ranked with the highest viability score.

### 2.3 Anticancer drug selection and calibration

All 10 anticancer drugs were selected based on their selective function and importance as drugs that are frequently used in the clinic. Ten drug concentrations were tested for each drug. hESC cells were grown on a 96 well plates in a triplicate manner. hESC density was 15,000 cells per well. For each drug, six plates were used to allow six time points per concentration. The drug in each concentration was added on Day ‘0’ and the medium was replaced every 24 h. Cell viability was assessed by a CellTiter‐Glo luminescent cell viability assay according to the manufacturer's instructions (Promega) and cell viability was monitored following 1, 2, 3, 6, 10 and 13 days. Luminescence reads for the target genes were normalized to control conditions, and the replicate experiments were averaged. This comprehensive calibration regime allowed a careful selection of concentrations that produce significant cell death yet allow some cell recovery. All anticancer drugs in this study: Azacytidine, Bleomycin, Vorinostat, Imatinib, Sunitinib, Vemurafenib, Methotrexate, Olaparib, Ibrutinib and Enzalutamide, were purchased from Cayman Chemical (Ann Arbor, Michigan, USA). Preparations of all drugs were done according to vendor protocols.

### 2.4 CRISPR screen of anticancer drugs resistant genes

Haploid hESC—CRISPR‐Cas9 LoF library cells were thawed on 10 cm Matrigel coated plates. Upon confluency, cells were harvested, counted and re‐seeded for each experiment. On the following day, control plates were harvested for DNA extraction and served as control. For the remaining plates, we replaced the medium with fresh mTeSR containing anticancer drugs in designated concentrations. Cell death and recovery were carefully monitored throughout the different experiments. Once cells recovered from the anticancer drug treatment, they were either harvested for DNA extraction or re‐seeded for an additional round of drug treatment and DNA extraction.

### 2.5 DNA extraction, PCR amplification of sgRNAs and high‐throughput DNA sequencing

Genomic DNA was extracted with a Blood & Cell Culture DNA Midi Kit (QIAGEN) or a gSYNC DNA extraction kit (Geneaid) according to the manufacturer instructions. The region containing the sgRNA integration was amplified with the following primers, which also contain overhang sequences compatible for Nextera DNA library preparations (Illumina):

5′‐TCGTCGGCAGCGTCAGATGTGTATAAGAGACAGGGCTTTATATATCTTGTGGAAAGGACG‐3′ (forward)
5′GTCTCGTGGGCTCGGAGATGTGTATAAGAGACAGACGGACTAGCCTTATTTTAACTTGC‐3′ (reverse),
Or
5′TCGTCGGCAGCGTCAGATGTGTATAAGAGACAGNNNNNNNNNNGGCTTTATATATCTTGTGGAAAGGACG 3′ (forward)
5′GTCTCGTGGGCTCGGAGATGTGTATAAGAGACAGACGGACTAGCCTTATTTTAACTTGC 3′ (reverse).

The total genomic DNA for each time point was divided into 50 μL PCR reactions with 4 μg DNA input. The PCR settings were as previously described.^18^ After purification of the 160‐base‐pair (bp) products, a second PCR reaction was performed using Nextera adapter primers to generate a Nextera DNA library according to the manufacturer's instructions (Illumina). DNA libraries containing sgRNA constructs from two replicate experiments were sequenced using NextSeq 500 (Illumina).

### 2.6 Data analysis

DNA next‐generation sequences were aligned to the sgRNA sequences using the bowtie2 programme^40^ allowing up to one mismatch. The count table was then normalized to the total number of reads in each of the specific drug concentrations, and replicates were averaged. CRISPR scores (CSs) are the average of the log_2_ ratios of the abundance of all sgRNAs for each gene between treated and control samples. Statistical significance was determined by the Kolmogorov–Smirnov test using ks_2samp from python's scipy. stats module as previously described.^18^ In doing so, the ratio of sgRNAs for every gene was compared between treated and control samples. The Benjamini–Hochberg False Detection Rate (FDR) correction was accomplished with the multiple tests feature from python's statsmodels.sandbox.stats.multicomp module.

### 2.7 Generation of knockout cell lines

1–3 sgRNAs per selected gene were used to target candidate genes for the anticancer drugs used in this study. sgRNA sequences for the genes *TET3*, *FLT4*, *PMAIP1* (*NOXA*), *PTEN* and *TP53* are summarized in Table S1. All sgRNAs were cloned into the lentiCRISPR v2 lentiviral vector (a gift from Feng Zhang, Addgene cat. no. 52961). To produce the lentiviruses, 293T cells were transfected with sgRNA‐containing lentiCRISPR v2 or sgRNA‐containing lentiCRISPR v2‐Blast, pCMV‐VSV‐G (a gift from Robert Weinberg, Addgene cat. no. 8454) and psPAX2 (a gift from Didier Trono, Addgene cat. no. 12260) plasmids at a ratio of 2:1:1.5 (10 mg total per plate), respectively, in the presence of polyethyleneimine ‘Max’ (PEI‐Max) (Polysciences) at a 1:2 ratio of DNA to PEI‐Max. Transfection medium was exchanged with standard hESC growth medium (described above) after 16–24 h, and lentiviral particle‐containing culture supernatant was harvested 60–65 h after transfection. Culture supernatant was spun down at 3000 rpm for 10 min at 4°C and then filtered through 0.45 mm cellulose acetate filters (Millipore). Filtered supernatant was frozen in aliquots at −70°C. Haploid‐enriched hESC cultures were trypsinized with Trypsin–EDTA, centrifuged and resuspended in hESC growth medium supplemented with 10 μM ROCK inhibitor Y‐27632 and 8 mg mL^−1^ polybrene (Sigma). The thawed viruses were then added to the cell suspension. Transduced cells were plated on feeder layer MEFs. Virus‐containing medium was replaced with standard hESC growth medium 24 h after transduction. The medium of the cells was replaced with medium that contains puromycin (0.3 mg/mL, Sigma) 36–48 h after transduction. Cells were kept under antibiotic selection for 7–10 days.

In *TP53* mutant cells, a GFP cassette was inserted to the end region of the tubulin gene using CRISPR–HOT method.^41^

Resistance/sensitivity assay was performed in 96 well plates, in triplicates in a similar format as the calibration assay. *TET3* KO cells were tested against azacytidine, *FLT4* (*VEGFR3*) KO cells were tested against sunitinib, *PMAIP1* (*NOXA*) KO cells were tested against vemurafenib and *PTEN* KO cell lines were tested against vorinostat, ibrutinib, enzalutamide and methotrexate.

### 2.8 Cell competition assay

In order to assess the resistance or insensitivity of TP53 LoF against various anticancer drugs, we applied the cell competition assay. In this assay, GFP‐labelled, TP53 KO cells were mixed in predetermined proportions with WT cells, and changes in the proportions of the labelled cells were measured by FACS analysis. The comparison of control plates (mixed cells with standard medium) versus drug‐treated plates enabled the distinction between a mutation growth advantage and response to drug treatment. TUBB::GFP TP53^−/−^ hESCs and WT hESCs were grown on Matrigel‐coated plates and harvested by TrypLE Select (Thermo Fisher Scientific) upon well confluency. WT and TUBB::GFP TP53^−/−^ cells were mixed in 98%:2% respectively, and were re‐seeded on 6‐well plates (Day −1). On Day 0, cells were harvested and prepared for FACS analysis (below). Control and experiment plates were kept with medium replacement (mTeSR, with or without anticancer drugs). The drugs tested are azacytidine, methotrexate and olaparib (Cayman Chemical, Ann Arbor, Michigan, USA) and the concentrations were as used in the genome‐wide screen. Cell competition monitoring was performed on Days 0, 5 and 9.

### 2.9 Flow cytometry

Cell samples for analysis of GFP expression were spun down at 1500 g for 4 min and the cell pellet was gently resuspended in 2 mL FACS solution (phosphate‐buffered saline with 2% FCS). Live cells were discriminated from cell debris and dead cells based on physical parameters (forward‐ and side‐light scatter). Fluorescence background levels were set with WT cells (GFP‐) cells. Following harvesting, cells were filtered through a 70 μm cell strainer and analysed by flow cytometry (BD Biosciences FACSAria III) and Flowjo software (FlowJo LLC).

### 2.10 Statistical analysis

Statistical analysis was performed using Python, R, JMP 15.0.0 software (SAS Institute Inc.) and Microsoft Office Excel. Data are presented as median‐centred. FDR was controlled using the Benjamini–Hochberg correction using an alpha of 0.05 for statistical significance.

## 3 RESULTS

### 3.1 Assessment of hESCs for cancer resistance screen

**FIGURE 1:**
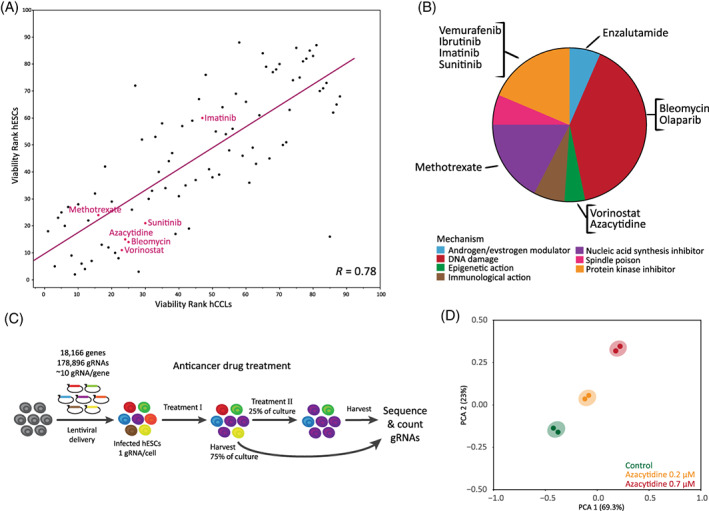
Evaluation and selection of anticancer drugs, genome‐wide screen via CRISPR/Cas9 knockout. (A) Human embryonic stem cell (hESC) and human cancer cell line (hCCL) response to anticancer drugs. The response of hESCs and hCCLs to 88 drugs from the approved oncology drug (AOD) set is presented. Cell response was ranked by survival following dosing (low rank = low survival). Of the 10 drugs included in this study, 6 were shared between these data sets, shown in red. (B) Pie chart of the AOD set divided by drug mechanism of action. The 10 drugs included in this study shown by mechanism. (C) Schematic illustration of the pooled CRISPR library screens used to identify resistance genes. (D) Principal component analysis (PCA) plot demonstrating the collected samples in the azacytidine screen.

To assess the relevance of hESCs for cancer resistance, we tested the response of hESCs to anticancer drugs from the AOD. hESCs were exposed to a range of AOD compounds and cell viability was previously measured.^42^, ^43^ We compared this viability data to the NCI‐60 Growth Inhibition Data. A total of 88 shared compounds were ranked by cell response from most to least potent and we then correlated our data with the NCI hCCLs' average response. Comparing hESCs to hCCLs (mean) provided a significant positive correlation (*R* = 0.78, *p* = 0.0012; Figure 1A), indicating the similarities in responses between hESCs and hCCLs to a given anticancer drug. Augmented by the knowledge about the molecular and cellular similarities between pluripotent cells and cancer cells (e.g., tumour formation capacity, telomerase activity, oncogene upregulation and high proliferation rate), the correlation suggests that human pluripotent cells may offer a platform for anticancer drug screening while avoiding the genomic complexity characterizing cancer cell lines.

### 3.2 Genome‐wide CRISPR LoF screen for anticancer drugs

**FIGURE 2:**
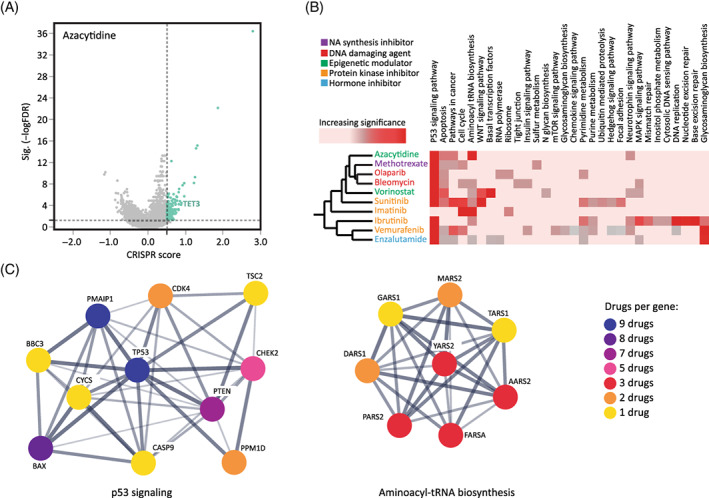
Analysis of human embryonic stem cell whole genome screens. (A) Volcano plot displaying the CRISPR score (CS = log2 fold change) and adjusted False Detection Rate (FDR) values for all the genes in the azacytidine screen. The threshold for enriched genes is designated by vertical and horizontal lines (CS >0.5, FDR <0.05, respectively). Green dots = significantly enriched genes. (B) Pathway enrichment of candidate genes. The top 100 genes for each of the 10 drugs were analysed for enrichment using the GSEA resource. Heatmap shows the KEGG pathway enrichment for each drug. Pink to red gradient = n.s. to highly significant (FDR) values. (C) Functional protein interaction network analyses using the STRING database. Genes from the P53 signalling pathway and the aminoacyl—tRNA biosynthesis pathway are presented showing shared and specific genes for the different drugs.

The field of anticancer drugs encompasses hundreds of compounds, widely varied in their mechanisms of action. We selected 10 different anticancer drugs for our screening (Figure 1B). Selection was based on drug mechanism (e.g., protein kinases, DNA damaging agents, etc.), their diverse cancer indications, and the presence of a drug in clinical use. Following selection of suitable drugs, we calibrated a dose response curve for each in order to select an optimal concentration for our genome‐wide LoF screens. The haploid hESC—CRISPR/Cas9 LoF library^18^ was exposed to the selected anticancer drugs in at least two different concentrations to allow both strong and moderate selection, capturing a wider snapshot of drug‐resistant genetic repertoire. We evaluated cell survival daily and once cells showed recovery, they were harvested for DNA extraction and replating at a ratio of 3:1, respectively. The replated cells were allowed to proliferate and then re‐exposed to the drug for further selection. By the end of the experiment, we sequenced 4–6 library samples alongside their respective controls (Figure 1C). Next, we analysed the changes in sgRNA abundance between the control and treatments via our CRISPR analysis pipeline.^18^, ^36^ Principal component analysis (PCA) was performed, showing clear and significant divergence of the treated samples from the controls in each of the 10 selected drugs. This effect was more pronounced for higher concentrations, later timepoints, or both (i.e., greater selective pressure; Figure 1D; Figure S1). A CS was calculated for every gene in our library by averaging the log_2_ ratio of abundance of all single guide RNAs (sgRNA) targeting each gene, comparing the drug‐treated samples and the respective control.^18^ Scanning compounds with a distinct mechanism of action using our whole‐genome LoF profiling can not only provide plausible drug resistance and critical candidate genes but may also reveal pathways involved in this resistance and its mechanism. A statistically significant and positive CS (CS > 0.5, FDR < 0.05) indicates enrichment of the mutant gene upon anticancer drug treatment, suggesting a possible drug‐resistant effect of the gene's null mutation. Upon quantifying the changes in abundance of mutants for all treated samples, we could identify drug resistance genes for all selected 10 drugs (Figure 2A; Figure S2). Furthermore, a careful comparison of all significant genes enabled us to define co‐shared genes (i.e., genes that might provide resistance to multiple tested compounds) alongside genes conferring resistance to a specific drug (Figure 2B,C). For example, the p53 signalling pathway was enriched in 9 of 10 drugs (analysing the top 100 genes for each drug; Table S2), whereas pathways such as aminoacyl—tRNA biosynthesis and basal transcription factors, were enriched in 7 of 10, or 2 drugs only (vorinostat and enzalutamide), respectively (FDR < 0.05; Figure 2B). The divergence of enriched pathways in our study demonstrated the applicability and the sensitivity of our haploid hESC screening system. Our comprehensive screens also allow deeper analysis within categories. We screened four protein kinase inhibitors; imatinib, sunitinib, vemurafenib and ibrutinib. Some are more specific tyrosine kinase inhibitors while others have a wider effect. Comparisons of the top 100 enriched genes for each drug resulted in a minimal overlap of four genes, and similar findings when performing dyad comparisons (Table S2). Pathway and gene ontology analysis on the non‐overlapping genes pointed to varied enrichments for each of the different drugs in this category.

### 3.3 Validation of candidate genes

**FIGURE 3:**
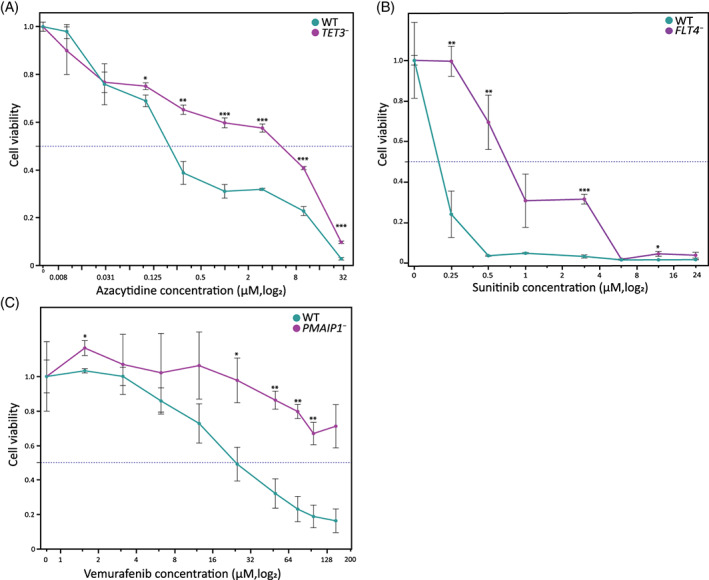
Individual validation of selected candidate genes. Candidate genes for three drugs were selected, knocked out, and their drug response compared to WT cells. (A) *TET3* ^*−*^ cells exhibit decreased sensitivity to azacytidine treatment. (B) *FLT4* ^*−*^ (VEGFR3) cells exhibit decreased sensitivity to sunitinib treatment. (C) *PMAIP1* ^*−*^ (NOXA) exhibit decreased sensitivity to vemurafenib treatment.

**FIGURE 4:**
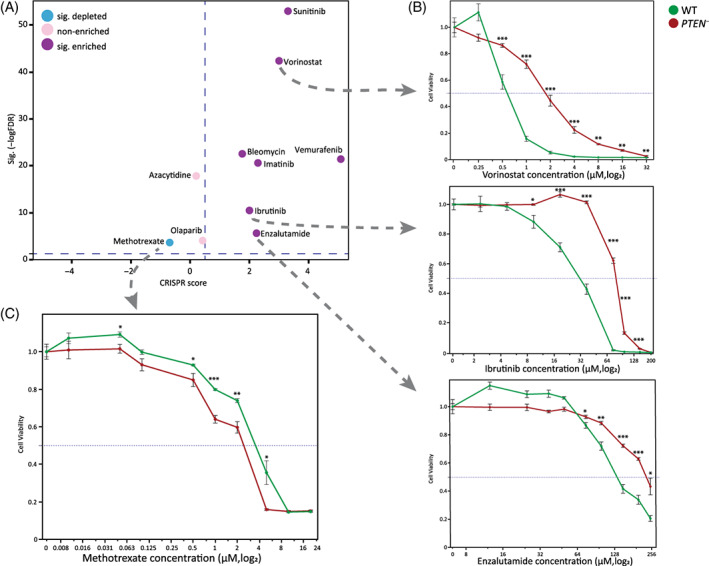
Effect of *PTEN* targeting on the response to anticancer drugs. The tumour suppressor *PTEN* was knocked out for validation on four drugs. (A) CRISPR score and False Detection Rate (FDR) values of *PTEN* ^*−*^ for all drugs included in this study, colour as in legend. (B) *PTEN* ^*−*^ cells exhibit decreased sensitivity to (from top) vorinostat (HDAC inhibitor), ibrutinib (tyrosine kinase inhibitor) and enzalutamide (androgen receptor antagonist). (C) *PTEN* ^*−*^ cells exhibit increased sensitivity to methotrexate (anti‐metabolite) treatment.

Our screens yielded a substantial number of plausible candidate genes. However, beyond the large screening analysis, individual validations are informative in specifically linking a gene's LoF mutation and resistance to a given drug. Our selection of candidate genes was based on their CS ranking, their molecular relevance to the drug's mechanism of action, and/or on the relevance of the candidate gene in cancer. We selected several candidate genes in which LoF mutations may confer resistance to anticancer drugs with diverse mechanisms of action. All selected candidate genes were confirmed as expressed in hESC as described.^18^ Azacytidine is given as a treatment for myelodysplastic syndromes and acute myeloid leukaemia. It has a known mechanism of action via inactivation of the de‐novo DNA methylase^44^ and indeed Tet Methylcytosine Dioxygenase 3 (*TET3*) was highly enriched in our screen (CS = 1.9, FDR = 0.0002; Figure 2A). Therefore, we selected the *TET3* gene for further validation. sgRNAs against *TET3* were designed and via the Lenti‐CAS9 vector system, *TET3* KO hESCs were generated. The mutated cells exhibited a dramatic decrease in sensitivity to azacytidine treatment (IC50 = 0.2 μm for WT and 5 μM for mutant; Figure 3A). Sunitinib acts as a cell signalling inhibitor by targeting tyrosine kinase receptors, primarily via the VEGF signalling pathway, which plays a role in both tumour angiogenesis and tumour cell proliferation.^45^ Three genes in this pathway were enriched in the sunitinib screen, among them Feline McDonough Sarcoma (FMS)‐Like Tyrosine Kinase 4 (*FLT4*, aka *VEGFR3*; CS = 1.7, FDR = 0.0002). An *FLT4* mutant line was generated and its response to sunitinib treatment evaluated. While WT hESCs exhibited sensitivity to sunitinib in low concentrations, the *FLT4* mutant cells were significantly insensitive (IC50 = 0.125 and 0.75 μM, respectively; Figure 3B). Vemurafenib is a selective BRAF kinase inhibitor, indicated for melanomas which possess a particular mutation (V600E, present in over half of cutaneous melanomas^46^). The gene Phorbol‐12‐Myristate‐13‐Acetate‐Induced Protein 1 (*PMAIP1*, aka *NOXA*) is a pivotal player in apoptotic pathways and although enrichment of it was found in all 10 drugs, it was significantly prominent in the vemurafenib screen (CS = 8.8, FDR = 4.5e‐25). Mutation in *PMAIP1* showed a dramatic resistance effect as the mutated hESCs were resistant to vemurafenib even in a high concentration (WT IC50 = 25 μM, *PMAIP1* ^*−*^ IC50 > 150 μM; Figure 3C). The Phosphatase And Tensin Homologue (*PTEN*) gene is a tumour suppressor that is frequently mutated in a large number of cancers,^47^ and as such makes an attractive candidate for screen validation. While *PTEN* was significantly enriched in 7 of the 10 screened drugs, it was significantly depleted in our methotrexate screen (CS = −1.8, FDR = 0.006; Figure 4A). For this validation assay, we selected three different drugs for which *PTEN* was enriched: Vorinostat (HDAC inhibitor), which is given as a treatment for cutaneous T cell lymphoma (CTCL^48^); Ibrutinib, a tyrosine kinase inhibitor used as a treatment for chronic lymphocytic leukaemia and various lymphomas^49^; Finally, enzalutamide is a relatively new anticancer drug used for the treatment of prostate cancer,^50^ via an androgen receptor (AR) signalling pathway. In the normal prostate, AR functions to balance proliferation vs. apoptosis rates and its expression in hESCs affects self‐renewal when inhibited.^51^ *PTEN* ^*−*^ hESCs showed decreased sensitivity to vorinostat with significant effect when comparing WT cells to the *PTEN* mutant cells (IC50 = 0.7 and 4 μM, respectively), as well as to ibrutinib (IC50 = 37 and 100 μM, respectively), and enzalutamide (IC50 = 125 and 250 μM, respectively; Figure 4B). Although positive selection screens such as ours are suboptimal for identifying drug‐sensitizing gene mutations (i.e., mutated genes depleted upon treatment), we speculated that given the significant depletion of *PTEN* ^*−*^ in the methotrexate screen, it might sensitize the mutant cells to the drug. Methotrexate is a veteran anticancer treatment which inhibits DHFR in the nucleic acid synthesis pathway and used to treat diverse cancer indications.^52^ In accordance with the screen results, *PTEN* mutation indeed made the hESCs more sensitive to methotrexate treatment compared to WT cells (IC50 = 2 and 4 μM, respectively; Figure 4C). Collectively, all individual validations exhibit the effectiveness of the whole‐genome screens.

To further support the relevance of the findings in genome‐wide screening in hESCs to actual tumours, we analysed cancer patient data from The Cancer Genome Atlas (TCGA). Most drugs included in this study had little to no data available between requiring drug treatment, genetic sequence, and tumour response details. However, methotrexate data was available for 19 patients, 7 resistant and 12 sensitive (defined as responsive or unresponsive in the TCGA). Looking at the top 20 enriched mutations from our screen in the patient data to compare prevalence of mutation in these genes in each group, 2 of 7 resistant patients showed mutation in one or more, compared to only 1 of 12 among the sensitive (29% vs. 8%). Upon including the top 100 genes (Table S2), 86% of the resistant patients had a mutation in at least one of the genes, while only 42% of sensitive patients had a mutation (proportion *Z*‐test; *p* = 0.081). Thus, the correlation between our identified genes to confer resistance to methotrexate and resistance in patients seems relatively significant, especially, when taking into account that many patients received methotrexate not as a monotherapy but as a combination of drugs.

### 3.4 *TP53* LoF effect on anticancer drug resistance

**FIGURE 5:**
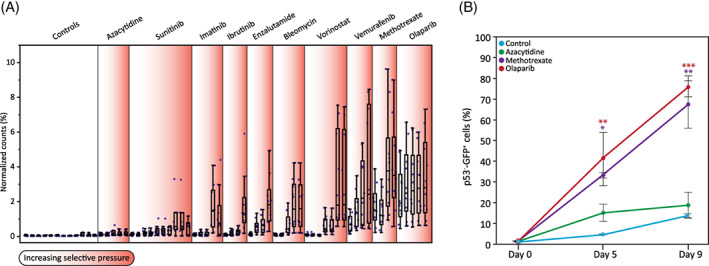
*TP53* knockout effect on drug resistance. *TP53* knockout exhibits a significant role in anticancer drug resistance. (A) Values of normalized *TP53* sgRNAs (%) are plotted for all samples included in this study. Black box plots represent the mean values of the normalized counts for each of the samples (control and treated plates). *X* axis = selective pressure, increasing duration and/or drug concentration per drug. (B) WT/*TP53* ^−/−^ cell competition assay. Mixture of WT and *TP53* ^−/−^ hESC were used to test *TP53* effect on drug resistance. Plates were seeded with 1:50 ratio of *TP53* ^−/−^:WT cells, then treated with either azacytidine, methotrexate, or olaparib. Changes in *TP53* ^−/−^–GFP hESC were measured by FACS. Used as designation of statistical significance, *p*‐value * < 0.05, ** < 0.01, *** < 0.001.

The importance of *TP53* in cancer with its pivotal and diverse roles in cell maintenance, growth, and survival is well documented.^53^ LoF mutations in *TP53* have been identified as such that allow cell survival and provide growth advantage in culture. Nevertheless, this growth‐advantage characteristic by itself is masking an effect that mutations in the *TP53* gene have on drug resistance. When we compared the *TP53* sgRNA reads (normalized as percent of all guides in the culture), we found dramatic differences among the 10 drugs tested (Figure 5A). While the controls did not exhibit a massive enrichment of *TP53* sgRNAs, this did occur in certain drug screens more than others (e.g., methotrexate and olaparib; Figure 5A). Interestingly, the enrichment of the *TP53* sgRNAs in the azacytidine assay was far less than for most of the other drugs. In order to address this interesting phenomenon and to distinguish between culture‐growth‐advantage per se and drug‐resistance effects, we generated a cell competition assay. As an elegant, easy‐to‐monitor system, we generated an hESC line with a LoF mutation in *TP53* and a tubulin‐tagged GFP fluorophore. Mixing the mutant cells with WT cells allowed for reliable detection in cell population dynamics over time and treatment. We tested four different conditions: control (medium only), azacytidine (antimetabolite and DNA methylation inhibitor), methotrexate (DHFR inhibitor), and olaparib (PARP inhibitor). The treated plates were dosed with drug concentrations like those used in the whole‐genome screens. Mixing WT and *TP53* mutant cells in a 98%:2% ratio, exhibited dramatic results (Figure 5B). While the control plates resulted in 10%–15% GFP+ cells in culture after 9 days, in either methotrexate‐ or olaparib‐treated plates, *TP53* mutant cells took over the culture (70%–80% GFP+ cells). Azacytidine‐treated cells resulted in only a mild increase of GFP+ cells compared to the control (GFP+ cells <20%). These dramatic differences demonstrate that *TP53* mutations are drug‐responsive and exhibit a phenotype far beyond the ‘classic’ growth advantage effect. In a similar analysis for a different member of the p53 pathway, *PMAIP1*(*NOXA*), we found no such global effect across screens (Figure S3), detecting a rise in sgRNA representation only for vemurafenib—which as previously mentioned was subsequently validated.

## 4 DISCUSSION

### 4.1 System efficacy

In this project, we established a system for using hESCs to identify mutations that may confer drug resistance. This platform is favourable to alternative approaches as it was established in haploid cells with a clean genetic background, which aids in the identification of specific genetic pathway activity. Along with the aforementioned common biology between ESCs and cancer cells, our comparison of response to anticancer drugs between our haploid hESCs and the CCL data (Figure 1) provided strong support for utilizing our hESCs in genome‐wide screen for drug‐resistant mutations. We selected 10 drugs arrayed across mechanisms of actions and indications that are actively used in cancer treatment. Our haploid hESC LoF library was exposed to these drugs and subsequent analysis enabled identification of potential resistance‐conferring mutated genes and the pathways involved.

### 4.2 Specificity of genes conferring resistance

In identifying the genes and pathways that allow resistance to the different anticancer drugs, various aspects of the selected genes align with previously known cellular effects of the drug, though it should be noted that our candidate genes have not been previously directly or specifically linked to resistance to these drugs. Among the enriched mutations in the azacytidine screen, and considering its known activity as a demethylating agent, we identified *TET3*, a gene encoding for a known demethylating enzyme. Sunitinib has known targets in VEGF receptors, and indeed we could identify *FLT4*/*VEGFR3* mutation as conferring resistance to the drug. In the case of vemurafenib, previous work has shown that *PMAIP1* is downregulated following inhibition of the BRAF pathway, the target of vemurafenib.^54^ We were able to show that lack of *PMAIP1*, a proapoptotic protein, grants a specific resistance to treatment with the drug.

### 4.3 Trends across multiple screens

Comparison of our results across the screens showed shared gene pathways among them. Mutations in the p53 signalling pathway showed a selective advantage against all 10 anticancer treatments. These include mutations in the gene encoding *TP53* itself, as well as the tumour suppressor *PTEN* (Figure 2C). Examination of *TP53* sgRNAs over time uncovered the differential impact of mutations in this gene on resistance for different anticancer drugs (Figure 5A). Azacytidine represents one side of the spectrum, showing increased representation of these mutants in a manner only slightly greater than in control samples. Several other drugs like imatinib or enzalutamide showed middling increased representation, especially when compared to the other extreme of response, where drugs such as methotrexate or olaparib showed well above half of the cells were *TP53* mutants. We could indeed validate the differential capacity of mutations in *TP53* to confer resistance to the drug (Figure 5B). These results support the power and sensitivity of our screen, as it detected a further level of gene‐drug response in addition to the well documented growth advantage of *TP53* mutants. As a final piece of supporting evidence, we were able to find TCGA data for a handful of bleomycin‐treated patients. When analysed for resistant mutations in treatment of bleomycin, among 53 patients with data available, 1 out of 3 resistant patients were mutated in *TP53*, while none of the 50 sensitive patients did. Proportion *Z*‐test between the groups showed *p* = 0.0263. This final piece of evidence regarding *TP53* supports the power of our screen.

Mutation of *PTEN*, also a key player in cancer and the p53 pathway, was utilized to perform validation screening for four different drugs across three mechanisms. *PTEN*'s prominent role as a tumour suppressing gene aligns with the increased drug resistance present in mutated cells. These finding are especially interesting for enzalutamide, which is indicated for prostate cancer, as *PTEN* is often mutated in this type of cancer, further implying the relevance of our screening process to cancer treatment practices. Our success in identifying a sensitivity to methotrexate in *PTEN‐*mutated cells was intriguing, and we suggest that perhaps we observed an interaction between growth advantage and increased proliferation from the mutation, and the inhibition of nucleotide synthesis by methotrexate causing increased cytotoxic effects on the cells.

Another pathway, not previously identified in the context of drug resistance, which was enriched across several screens was the aminoacyl‐tRNA biosynthesis pathway, responsible for attaching tRNA to its respective amino acid preceding translation during gene expression (Figure 2C). While these genes were enriched in several drug screens, the strongest effect (and greatest pathway representation) was seen in the azacytidine screen. While a mechanistic interpretation of these findings is outside the scope of the current work, we suggest that perhaps as azacytidine causes demethylation and increased gene transcription, damage to tRNA production helps curb the perturbation as the cell's resources are misspent, conversely assisting survival in cells with a mutated pathway.

Our screen includes four protein kinase inhibitor drugs: Sunitinib, imatinib, vemurafenib and ibrutinib. Despite their shared mechanism, their actual target proteins and the roles of those targets in their pathways vary, therefore variation in enriched genes is expected. Analysis of significantly enriched genes indeed yielded different gene groups with a minimal overlap between all four drugs. Predictably, the overlap genes are all part of the p53 pathway, and all four drugs exhibit enrichment for cell cycle and cellular response to DNA damage stimulus.

This work demonstrated our established system to be robust, enabling identification of genes that confer resistance to the tested drugs. However, we selected a sampling of candidate genes to validate the genome‐wide results, and not every gene detected was validated. Candidate genes were mostly chosen based on their known function, and additional novel candidates can be tested in the future. Still, even the known tumour suppressor gene, *PTEN*, has not been previously reported as granting drug resistance, and its effect was validated in four drugs (three for resistance and one sensitivity; Figure 3). Moreover, the genes validated in our study have not been previously confirmed as resistance‐granting to anticancer treatment.

It should also be acknowledged that current drug treatments for cancer are overwhelmingly administered in combinations of multiple drugs designed for maximum efficacy, a fact not addressed by our single‐agent screens. While initial drug selection attempted to account for this (i.e., selection of drugs also used as monotherapies), clinical practices of combination therapy will require further elaboration of screens in our system to address this point.

### 4.4 Genome‐wide screens

As mentioned above, work examining drug response in cancer cell lines has been previously performed.^6^ Databases similar to the TCGA have been established covering gene‐drug interactions among numerous other biological properties of several hundreds of cancer cell lines, though as stated the genetic stability of these cells is suboptimal.^55^ Genome‐wide CRISPR screens in hundreds of hCCLs have been performed with the purpose of optimizing drug treatment through treatment protocol prioritization.^56^ However, CRISPR‐Cas9 has been shown to be affected by copy number aberrations, which are very prevalent in cancers, skewing and even invalidating results.^57^ Research utilizing a genome‐wide CRISPR approach on the nearly‐haploid CCLs HAP1 and KBM7^58^ would bypass this interference, however such work is still plagued by the aforementioned chromosomal instability,^23^ in addition to point mutations,^18^ leading to difficulty in analysing and interpreting results. Our screen takes a novel approach, synergizing the utility of healthy cells with a clean genetic background and true haploidy, enabling the full benefit of this method to be brought to bear with greater capacity for detection, indicating multiple pathways and genes not previously identified for the drugs examined here. For example, the effects of the p53 pathway shown here would likely be masked in hCCLs without these advantages. Collectively, this work has shown strong evidence for the power, sensitivity and advantage of our resistance‐screening system, and should complement existing knowledge in the furtherance of efficient treatment of cancer.

## AUTHOR CONTRIBUTIONS

Emanuel Segal, Jonathan Nissenbaum, Mordecai Peretz, Oded Kopper and Nissim Benvenisty designed the experiments; Emanuel Segal, Jonathan Nissenbaum and Mordecai Peretz performed the experiments with assistance from Tamar Golan‐Lev and Rivki Cashman; Hagit Philip assisted with the statistical analysis; Emanuel Segal and Jonathan Nissenbaum wrote the manuscript with input from Benjamin E. Reubinoff, Oded Kopper and Nissim Benvenisty; Oded Kopper and Nissim Benvenisty supervised the work.

## FUNDING INFORMATION

We thank members of The Azrieli Center for Stem Cells and Genetic Research for critical reading of the manuscript. The results published or shown here are in whole or part based upon data generated by the TCGA Research Network: https://www.cancer.gov/tcga. This work was partially supported by the Azrieli Foundation, the Israel Science Foundation (2054/22), the ISF‐Israel Precision Medicine Partnership (IPMP) Program (3605/21) and by NewStem Ltd. Nissim Benvenisty is the Herbert Cohn Chair in Cancer Research.

## CONFLICT OF INTEREST STATEMENT

Oded Kopper and Nissim Benvenisty are V.P. R&D and CSO of NewStem Ltd, respectively.

## Supporting information


**Figure S1:** Principal component analysis (PCA) plots of the different samples collected per drug. (A–I) PCA plot showing the collected samples for each remaining drug screen (azacytidine shown in Figure 1). ‘a’ and ‘b’ are single and repeated exposure to the drug, respectively.
**Figure S2:** Volcano plots of the CRISPR Scores for nine drugs. (A–I) Volcano plots showing the CS for screening of nine drugs (azacytidine is shown in Figure 2). Plots generated by data normalization and flooring to account for fully depleted gRNA counts.
**Figure S3:** *PMAIP1* knockout effect on drug resistance. *PMAIP1* (NOXA) sgRNA enrichment across all samples for all screens.

**Table S1:** sgRNA sequences for generation of knockout cells for *TET3*, *FLT4*, *PMAIP1* (*NOXA*), *PTEN* and *TP53*.

**Table S2:** Top 100 enriched mutated genes for all 10 anticancer drugs in our drug resistance screen, ranked by CRISPR score.

## Data Availability

The data that support the findings of this study are available from the corresponding authors upon reasonable request.
