# Supplementary material for: Genome‐wide screen for anticancer drug resistance in haploid human embryonic stem cells
Source: Cell Prolif. 2023 Apr 21;56(6):e13475. doi: 10.1111/cpr.13475 (PMC10280149; doi:10.1111/cpr.13475)
Supplement: Supplementary file 1 — Figure S1: Principal component analysis (PCA) plots of the different samples collected per drug. (A–I) PCA plot showing the collected samples for each remaining drug screen (azacytidine shown in Figure 1). ‘a’ and ‘b’ are single and repeated exposure to the drug, respectively. Figure S2: Volcano plots of the CRISPR Scores for nine drugs. (A–I) Volcano plots showing the CS for screening of nine drugs (azacytidine is shown in Figure 2). Plots generated by data normalization and flooring to account for fully depleted gRNA counts. Figure S3: PMAIP1 knockout effect on drug resistance. PMAIP1 (NOXA) sgRNA enrichment across all samples for all screens. [file CPR-56-e13475-s002.docx]

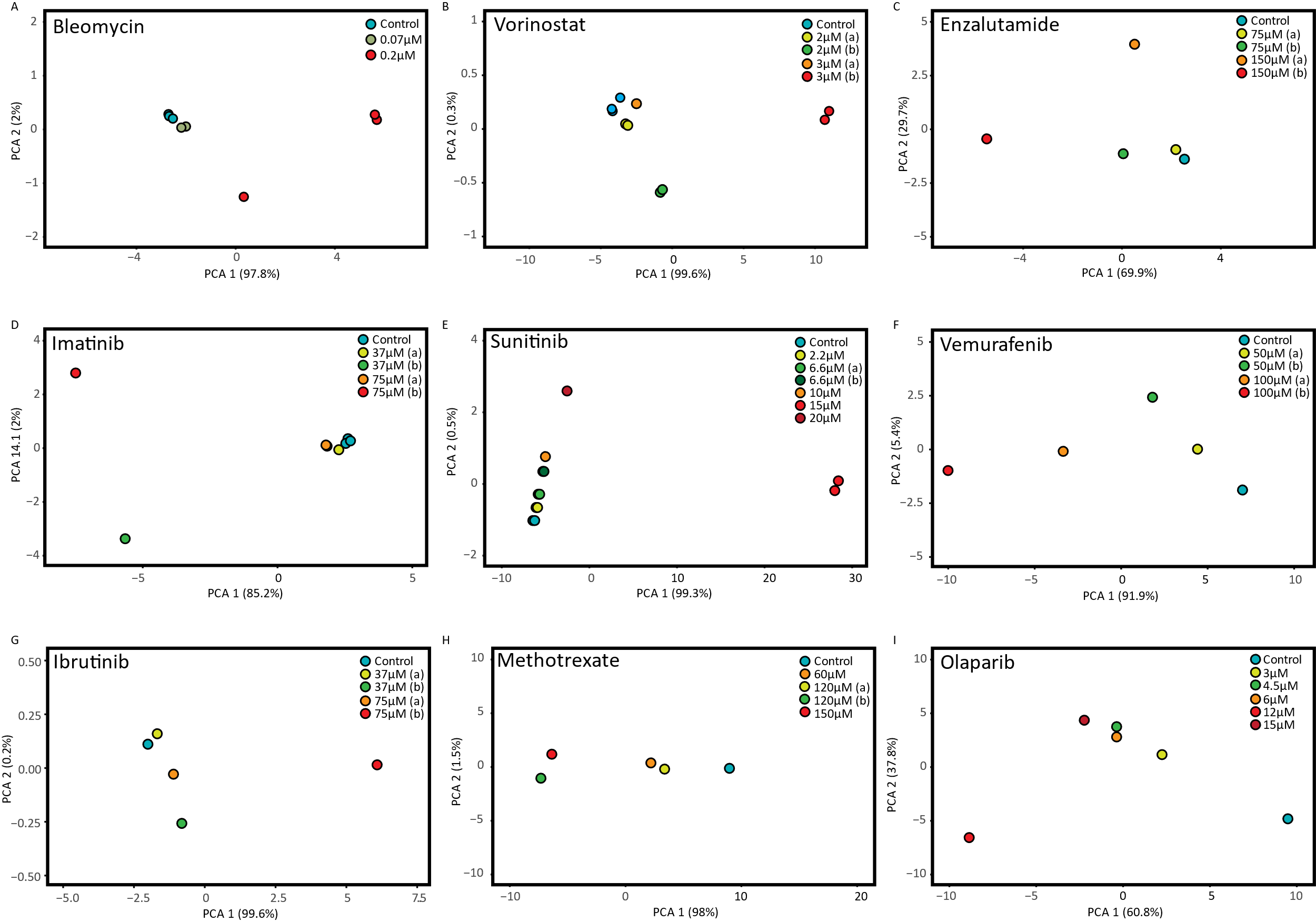
**Figure S1: PCA plots of the different samples collected per drug**

**A-I.** PCA plot showing the collected samples for each remaining drug screen (azacytidine shown in Figure 1). “a” and “b” are single and repeated exposure to the drug, respectively.


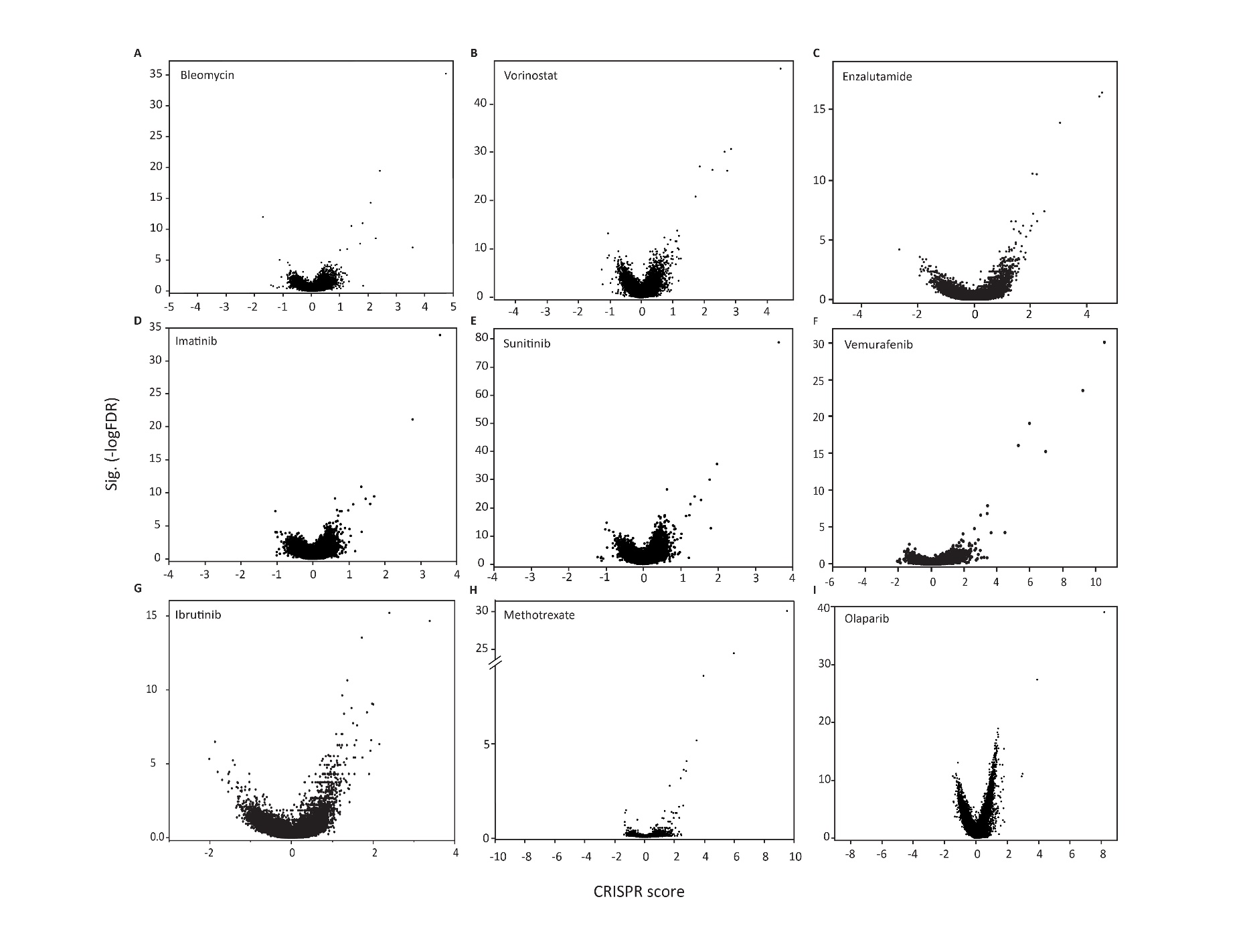
**Figure S2: Volcano plots of the CRISPR Scores for nine drugs**

**A-I.** Volcano plots showing the CS for screening of nine drugs (azacytidine is shown in Figure 2). Plots generated by data normalization and flooring to account for fully depleted gRNA counts.


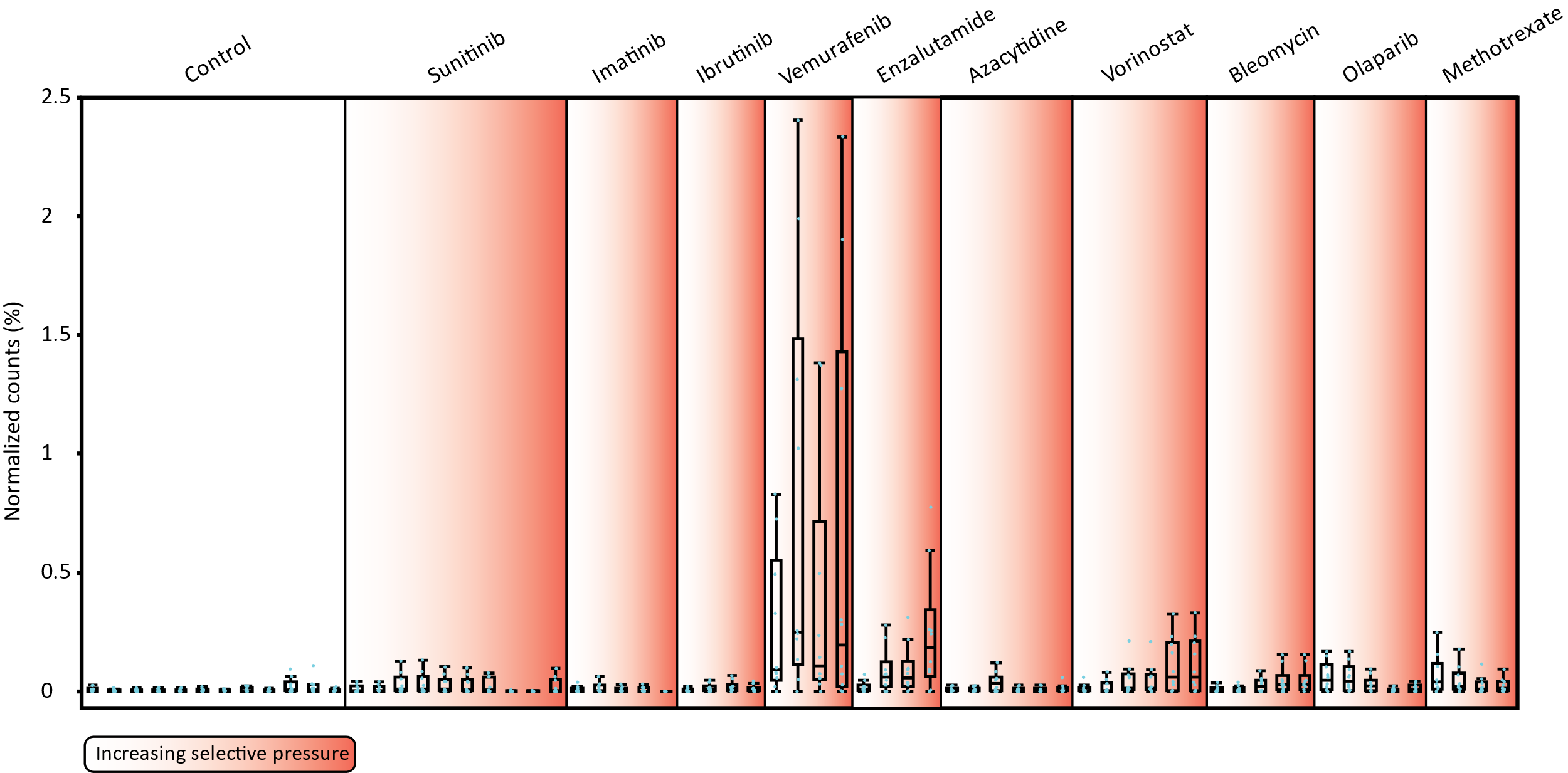
**Figure S3: *PMAIP1* knockout effect on drug resistance**

*PMAIP1* (NOXA) sgRNA enrichment across all samples for all screens.
